# Effects of local structure of neuronal networks on spiking activity *in silico*

**DOI:** 10.1186/1471-2202-12-S1-P202

**Published:** 2011-07-18

**Authors:** Tuomo Mäki-Marttunen, Jugoslava AćimoviAć, Keijo Ruohonen, Marja-Leena Linne

**Affiliations:** 1 Department of Signal Processing, Tampere University of Technology, Finland; 2 Department of Mathematics, Tampere University of Technology, Finland

## 

The structure of the neuronal network, including synaptic connectivity, is the basis for information transfer in the network. Various graph-theoretic measures such as degree distribution, mean geodesic path length, clustering coefficient and motif distribution exist for analysing the structure of networks [1], and each of them captures only one perspective of the properties that are crucial regarding the activity in the network. In this work, we vary the local structure of neuronal networks and observe changes in their activity *in silico*, i.e. in simulations where the activity of single neurons and their interaction is modeled. The local structure is analysed through the occurrence of different motifs, i.e. different patterns of connectivity. The effect of motifs on network dynamics has been widely studied in different types of networks: from the stability point of view in networks with unspecified dynamics [2], in artificial neural networks [3], and from synchronization point of view in spiking neuronal networks [4]. Our work focuses on noise-driven neuronal networks, where the activity can be characterised by spike trains of neurons in the network, and particularly by the bursting behaviour of the network.

**Figure 1:**
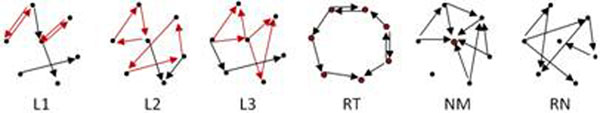
Different network structures. Illustration of different networks used in the present work. The red arrows and balls represent characteristics of each network, which are the following: Bidirectional edges in L1, loops of length 3 in L2, feed-forward triples in L3, nodes being placed in a ring in RT and connected to nearest neighbors, and possibility of nodes with high in-degree in NM.

To study the local structure of networks we consider the occurrences of three separate connectivity patterns: (1) the bidirectional edges, (2) the loops of three nodes, and (3) the feed-forward motifs of triples of nodes. Networks with one of these three local connectivity patterns promoted are generated – we abbreviate these networks (L1), (L2) and (L3). In addition, different distance-dependent networks are generated, including networks with ring topology (RT) and biologically plausible topology, obtained by the NETMORPH [5] simulator (NM). All networks except for NM have binomially distributed in-degree, as is the case with the random networks (RN) that are widely used in neuronal activity simulations. Small illustrations of these network structures are shown in Figure 1. Neuronal activity in these types of networks of size N=100 is simulated using the model presented in [6]. The simulations show a difference in the activity of these networks. Preliminary results indicate, that network bursts occur more frequently in distance dependent networks RT and NM, especially in RT. Accordingly, the overall spiking frequency is high in these networks, but also in L3 networks.
